# Potential Cardiovascular Risk Protection of Bilirubin in End-Stage Renal Disease Patients under Hemodialysis

**DOI:** 10.1155/2014/175286

**Published:** 2014-09-10

**Authors:** Maria do Sameiro-Faria, Michaela Kohlova, Sandra Ribeiro, Petronila Rocha-Pereira, Laetitia Teixeira, Henrique Nascimento, Flávio Reis, Vasco Miranda, Elsa Bronze-da-Rocha, Alexandre Quintanilha, Luís Belo, Elísio Costa, Alice Santos-Silva

**Affiliations:** ^1^Instituto de Ciências Biomédicas Abel Salazar, Universidade do Porto, Rua Jorge Viterbo Ferreira 228, 4050-313 Porto, Portugal; ^2^Frenesius Medical Care, Nephrocare Maia, SA, Rua Altos 70, Maia, 4470-235 Porto, Portugal; ^3^Laboratório de Bioquímica, Departamento de Ciências Biológicas, Faculdade Farmácia, Universidade do Porto, Rua Jorge Viterbo Ferreira 228, 4050-313 Porto, Portugal; ^4^Instituto de Biologia Molecular e Celular (IBMC), Universidade do Porto, Rua do Campo Alegre 823, 4150-180 Porto, Portugal; ^5^Centro Investigação Ciências Saúde, Universidade Beira Interior, Covilhã, Avenida Infante D. Henrique, 6200-506 Covilhã, Portugal; ^6^IBILI, Faculdade de Medicina, Universidade de Coimbra, Azinhaga Santa Comba, Celas, 3000-548 Coimbra, Portugal

## Abstract

We evaluated the potential cardiovascular risk protection of bilirubin in hemodialysis (HD) patients. An enlarged set of studies were evaluated in 191 HD patients, including hematological study, lipid profile, iron metabolism, nutritional, inflammatory markers, and dialysis adequacy. The TA duplication screening in the UDP-glucuronosyltransferase 1 A1 (*UGT1A1*) promoter region was also performed. The *UGT1A1* genotype frequencies in HD patients were 49.2%, 42.4%, and 8.4% for 6/6, 6/7, and 7/7 genotypes, respectively. Although no difference was found in *UGT1A1* genotype distribution between the three tertiles of bilirubin, significant differences were found with increasing bilirubin levels, namely, a decrease in platelet, leukocyte, and lymphocyte counts, transferrin, oxidized low-density lipoprotein (ox-LDL), ox-LDL/low-density lipoprotein cholesterol ratio, apolipoprotein (Apo) A, Apo B, and interleukin-6 serum levels and a significant increased concentration of hemoglobin, hematocrit, erythrocyte count, iron, transferrin saturation, Apo A/Apo B ratio, adiponectin, and paraoxonase 1 serum levels. After adjustment for age these results remained significant. Our data suggest that higher bilirubin levels are associated with beneficial effects in HD patients, by improving lipid profile and reducing the inflammatory grade, which might contribute to increase in iron availability. These results suggest a potential cardiovascular risk protection of bilirubin in HD patients.

## 1. Introduction

Despite the technological advances in hemodialysis (HD) procedures and medical support in the last years, the mortality and morbidity of end-stage renal disease (ESRD) patients under HD remain 10 to 20 times higher than those observed in the general population [1–3]. Cardiovascular disease (CVD) events are the main cause of death in these patients [2]. The prevalence of the classic cardiovascular risk factors, namely, hypertension, diabetes* mellitus*, dyslipidemia, smoking habits, and advanced age* per se,* cannot explain the cardiovascular mortality rate. The enhanced inflammatory response and oxidative stress usually observed in HD patients or even other unknown factors may, therefore, play an important role in the cardiovascular morbidity and mortality rate in ESRD patients under HD [4].

In 1987, bilirubin was proposed as a potential physiological antioxidant and anti-inflammatory agent by Stocker et al.;* they* showed that bilirubin, at physiological concentrations, protects cell membrane fatty acids from oxidation by peroxyl radicals [5]. It has been shown that both the unconjugated and conjugated forms of bilirubin can protect low-density lipoprotein cholesterol (LDL-c) and other lipids from oxidation by reactive oxygen species [6–8], leading to reduced baseline levels of oxidized LDL, especially in individuals with higher bilirubin levels [9]. Several other* in vitro* and* in vivo* studies [5, 10–14] showed bilirubin as an antioxidant and, therefore, as an important factor in tissue protection against oxidative and inflammatory damage [12, 15, 16].

Bilirubin is a water-insoluble compound that requires glucuronidation by a microsomal enzyme, the uridine diphosphate glucuronosyltransferase-1 A1 (UGT1A1), to be excreted. The* UGT1A1 locus* has been mapped to chromosome 2q37 [17] and one of the most common genetic variants that affects the glucuronidation of bilirubin in Caucasians is a TA duplication polymorphism in the TATA box region of the promoter. Homozygous individuals carrying the A(TA)7TAA allele (c.-41_-40dupTA or [TA]7) have higher levels of unconjugated bilirubin, caused by a reduction of 30% in the* UGT1A1* transcription [17]. There are few studies on the effect of bilirubin levels and/or of* UGT1A1* gene polymorphism in the outcome of CVD in the general population, namely, in the development of coronary artery disease, coronary heart disease, peripheral vascular disease, and stroke. Recent epidemiological evidences showed a reduced incidence of lung disease and all-cause mortality in individuals with high serum bilirubin levels and with Gilbert's syndrome [18–20]. Moreover, a study evaluating the impact of bilirubin levels and of* UGT1A1* polymorphisms on CVD risk and mortality in ESRD under HD [21] showed that HD patients with lower serum bilirubin levels presented a more adverse outcome and, therefore, that the 7/7 genotype might have an important effect on preventing CVD events and death. Nevertheless, the mechanisms underlying this protective effect of bilirubin, in the general population and in ESRD patients, still remain obscure. Multiple mechanisms could explain the protective effect of bilirubin, including antioxidant and anti-inflammatory pathways, which may be related to the powerful redox cycle mediated by biliverdin reductase that may protect against pathological oxidation processes occurring during cardiovascular disease [22].

In this work, we aimed to evaluate the potential cardiovascular risk protection of bilirubin in ESRD patients under HD. For this, clinical and sociodemographic data, lipid profile, hematological, dialysis adequacy, inflammatory and iron metabolism markers, and screening for the TA duplication in the TATA box of the* UGT1A1* promoter were studied in an ESRD Portuguese sample.

## 2. Material and Methods

### 2.1. Subjects

This transversal study included 191 ESRD Portuguese patients under HD (105 males and 86 females, mean age: 66.13 years; standard derivation [SD]: 14.02 years). All participants gave their informed consent to participate in this study that was previously approved by the Ethics Committee of Fresenius Medical Care, Portugal.

Patients with malignancy, autoimmune disease, and inflammatory or infectious diseases and with increased levels of alanina transaminase and/or aspartate transaminase were excluded. Patients were under therapeutic HD three times per week, 3–5 hours each session, for a median time of 2.13 (0.82–5.24) years, with a dose of darbepoetin-*α* of 0.4 (0.2–0.7) *μ*g/kg/week. For the HD procedure, high-flux polysulfone FX-class dialyzer of Fresenius (Bad Hamburg, Germany) was used. The main causes of renal failure in our patients were diabetic nephropathy (*n* = 69), hypertensive nephrosclerosis (*n* = 22), nephritic syndrome (*n* = 10), polycystic kidney disease (*n* = 8), obstructive diseases (*n* = 7), hereditary nephropathy (*n* = 3), chronic interstitial nephritis (*n* = 2), benign prostate hypertrophy (*n* = 1), other diseases (*n* = 8), and uncertain etiology (*n* = 61). The Kt/V urea was calculated using standard formula that takes into consideration the postdialysis serum urea nitrogen concentration and compares this with the initial or predialysis level.

Blood was collected immediately before the HD procedure, on the second dialysis session of the week, into tubes containing ethylenediaminetetraacetic acid (EDTA) and into tubes without anticoagulant, in order to obtain whole blood, serum, and plasma. Blood samples were processed within 2 hours of collection. Aliquots of plasma, serum, and buffy coat were immediately stored at −80°C until the assays were performed.

### 2.2. Hematologic and Biochemical Assays

Platelet, leukocyte, and erythrocyte counts and hematocrit and hemoglobin concentration were measured by using an automatic blood cell counter (Sysmex K1000; Sysmex, Hamburg, Germany). Differential leukocyte counts were evaluated in Wright-stained blood smears. Reticulocyte count was made by microscopic counting on blood smears after vital staining with new methylene blue (reticulocyte stain; Sigma, St. Louis, MO, USA). The reticulocyte production index (RPI) was calculated as an appropriate way to measure the effective erythrocyte production, by correcting for both changes in hematocrit (degree of anemia) and for premature reticulocyte release from the bone marrow [22]. Total bilirubin (TB) was evaluated using a commercially available kit (diazotized sulfanilic acid reaction, Roche Diagnostic).

Serum iron concentration was determined using a colorimetric method (Iron, Randox Laboratories Ltd., North Ireland, UK), whereas serum ferritin and transferrin were measured by immunoturbidimetry (Ferritin, Laboratories Ltd., North Ireland, UK; Transferrin, Laboratories Ltd., North Ireland, UK). Transferrin saturation (TS) was calculated by the formula: TS(%) = 70.9 × serum iron concentration in *μ*g/dL/serum transferrin concentration in mg/dL. Enzyme-linked immunosorbent assays were used to measure serum soluble transferrin receptor (sTfR; human sTfR immunoassay, R&D Systems, Minneapolis, MN, USA). Plasma levels of hepcidin-25 were quantified using a peptide enzyme immunoassay (Bachem Group, Peninsula Laboratories, LLC, San Carlos, California).

The lipid profile was performed in an autoanalyzer (Cobas Mira S, Roche, Basel, Switzerland), using commercially available kits; total cholesterol and triglycerides concentrations were determined by enzymatic colorimetric tests (cholesterol oxidase-phenol aminophenazone and glycerol-3-phosphate oxidase-phenol aminophenazone methods, Roche, resp.); high-density lipoprotein cholesterol (HDL-c) and LDL-c were measured using enzymatic colorimetric tests, after selective separation of HDLc and LDLc fractions (direct HDL cholesterol and direct LDL cholesterol, Roche, resp.); oxidized LDL (Ox-LDL) was measured directly in plasma by using a two-site enzyme immunoassay (oxidized LDL ELISA, Mercodia, Uppsala, Sweden); serum levels of apolipoprotein A-I (Apo A-I) and Apo B were evaluated by immunoturbidimetric assays (unikit apolipoprotein A-I and B specific antiserums, Roche); serum lipoprotein(a) [Lp(a)] was quantified by using an immunoturbidimetric method (Lp(a) Roche Diagnostics).

Serum C-reactive protein (CRP) was determined by nephelometry [CRP (latex) high-sensitivity, Roche Diagnostics] and serum interleukin-6 (IL-6) was evaluated by enzyme immunoassays (human IL-6 high-sensitivity ELISA, eBioscience, Vienna, Austria). Serum albumin levels were measured using a colorimetric assay end-point method (albumin plus; Roche GmbH, Mannheim, Germany). Plasma levels of adiponectin were evaluated by using a standard commercial enzyme-linked immunoassay (Bender MedSystems, San Diego, CA, USA).

The activity of paraoxonase 1 (PON1) was assessed spectrophotometrically and expressed in nmol of p-nitrophenol/mL/min. Briefly, paraoxonase activity was measured by adding serum to 1 mL Tris/HCl buffer (100 mmol/L, pH 8.0) containing 2 mmol/L CaCl_2_ and 5.5 mmol/L paraoxon (O,O-diethyl-O-p-nitrophenylphosphate; Sigma Chemical Co.). The rate of generation of p-nitrophenol was determined by reading the absorbance at 412 nm, at 37°C, with the use of a continuously recording spectrophotometer (Beckman DU-68).

### 2.3. DNA Analysis

Genomic DNA was extracted from white blood cells (buffy coat) by proteinase K/salt precipitation method. Genotyping TA duplication in the TATA box of the* UGT1A1* promoter was performed by polymerase chain reaction (PCR). Amplification reaction was carried out in a thermocycler (MiniOpticon Real-Time PCR Detection System; Biorad) using 2 *μ*L of DNA, 0.5 *μ*L of each primer at a concentration of 10 pmol (forward: 5′-TAACTTGGTGTATCGATTGGTTTTTG-3′; reverse: 5′-ACAGCCATGGCGCCTTTGCT-3′) and 7.5 *μ*L of PCR Master Mix Promega (M750B) and water for a final volume of 15 *μ*L. The first step of PCR was 95°C denaturation for 5 minutes, followed by 35 cycles: denaturation at 95°C for 30 seconds, annealing at 55°C for 30 seconds, extension at 72°C for 45 seconds, and final extension at 72°C for 10 minutes. PCR was followed by electrophoresis in 15% polyacrylamide gel in a Tris/borate/EDTA buffer; the gel was stained with silver nitrate and photographed.

### 2.4. Statistical Analysis

Statistical analysis was performed using the Statistical Package for Social Sciences (SPSS, version 21.0) for Windows (SPSS Inc., Armonk, NY, USA). The normal distribution of continuous variables was analyzed using the Kolmogorov-Smirnov test. Continuous variables without normal distribution were log transformed. ANOVA, supplemented with Tukey's HSD* post hoc* test, was conducted in order to evaluate the effect of tertiles of bilirubin on continuous covariates. ANCOVA analysis was performed for age adjustment. The association between tertiles of bilirubin and categorical variables was analyzed using the Chi-squared test or Fisher's exact test. Pearson's rank correlation coefficient was used to evaluate relationships between sets of data. The significance level (*α*) was set at 0.05.

## 3. Results

The results were analyzed by two ways, according to the* UGT1A1* genotype, in order to evaluate the changes associated with a decreasing* UGT1A1* activity, and in accordance with bilirubin levels. For the second analysis, we used the tertiles of TB as follows: TB < 0.21 mg/dL (T1), TB 0.21–0.3 mg/dL (T2), and TB > 0.3 mg/dL (T3).

**Figure 1:**
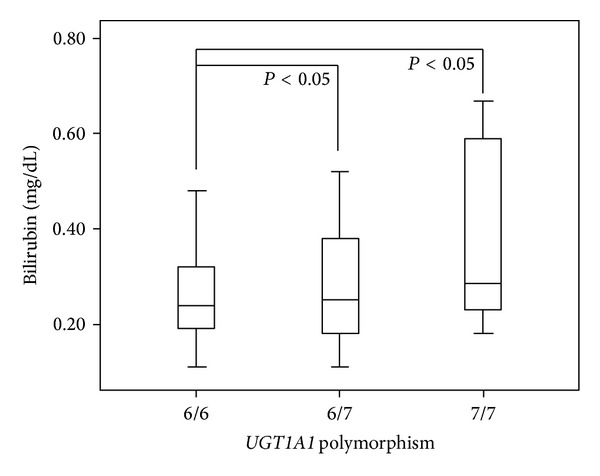
Total bilirubin levels in ESRD patients under HD according to the number of TA repeats in the promoter region of* UGT1A1*.

The* UGT1A1* genotype frequencies in our HD patients were 49.2%, 42.4%, and 8.4% for 6/6, 6/7, and 7/7 genotypes, respectively. When we stratified the results according to the* UGT1A1* genotype, we found that HD patients with the 7/7 genotype presented significantly higher TB levels than patients with 6/6 genotype and that patients with the 6/7 genotype showed significantly higher TB levels than patients with 6/6 genotype (Figure 1). The HD patients with the 7/7 genotype presented also a significant increase in HDL-c (6/6 genotype: 38.5 ± 13.4 mg/dL; 6/7 genotype: 37.0 ± 12.4 mg/dL; 7/7 genotype: 52.7 ± 17.0 mg/dL; *P* < 0.05, 7/7 genotype versus 6/6 and 6/7 genotypes). Beyond the increase in HDL-c and bilirubin in HD patients with 7/7 genotype, no additional significant differences were observed for the other studied variables.

When performing multiple comparisons between the bilirubin tertile groups (Table 1), we observed lower values of platelets, white blood cells, and lymphocytes in HD patients in the third tertile group. Concerning the iron metabolism, this third tertile group showed increasing values in iron and transferrin saturation and decreasing values in transferrin. In the lipid profile, lower values were found for ox-LDL, ox-LDL/LDLc ratio, Lp(a), Apo A, and Apo B and higher values for Apo A/Apo B ratio in the third tertile compared to the first and second tertiles groups. Increasing values of adiponectin and PON1 were also observed in the third tertile group.* Post hoc* analysis showed that HD patients in the second tertile of bilirubin, as compared with those in the first tertile, presented a significant increase in hemoglobin concentration, hematocrit, and erythrocyte count; a significant decrease was also observed for ox-LDL/LDLc ratio, Apo B, and IL-6. Comparing HD patients in the third tertile of bilirubin with those in the first tertile, we found a significant reduction in platelet, leukocyte, and lymphocyte counts; iron metabolism presented significant changes, namely, an increase in serum iron and in transferrin saturation, and a decrease in transferrin; several significant changes were also observed in the lipid profile, namely, a significant decrease in ox-LDL, ox-LDL/LDLc ratio, Apo A, and Apo B and a significant increase in Apo A/Apo B ratio; for the inflammatory markers, we found a significant rise in adiponectin and PON1 and a significant reduction in IL-6 serum levels. After adjusting for age, the results remained significant (Table 1). No statistical significant differences were found in* UGT1A1* genotype between the three tertiles of TB, although HD patients homozygous for the (TA)7 allele showed an increase in bilirubin levels when compared to those with genotypes 6/7 and 6/6. Additionally, applying Pearson's rank correlation, we also found statistically significant correlations between TB and adiponectin (*r* = 0.238; *P* = 0.001), transferrin (*r* = −0.213; *P* = 0.003), iron (*r* = 0.201; *P* = 0.005), transferrin saturation (*r* = 0.307; *P* < 0.001), ferritin (*r* = 0.173; *P* = 0.017), Apo A (*r* = −0.249; *P* < 0.001), lymphocytes (*r* = −0.223; *P* = 0.002), and IL-6 (*r* = −0.193; *P* = 0.008).

## 4. Discussion

Bilirubin is a key metabolic product of hemoglobin catabolism and seems to have a protective effect in oxidative stress conditions, such as atherosclerosis, coronary heart disease, inflammatory diseases, cancer, and renal disease [8, 21, 23, 24]. Plasma bilirubin concentrations higher than 20 mg/mL are associated with deleterious effects in fetus and newborns, by increasing the risk of neurological dysfunction [25, 26], as a result of its toxic effect on neuronal tissue. Under physiological conditions, most of the bilirubin is bound to albumin. Homozygosis for the TA duplication in the promoter region of* UGT1A1* gene, associated with higher levels of unconjugated bilirubin, is considered as the main cause of Gilbert syndrome in Caucasian population [2, 17] and justifies some of the interindividual variations in bilirubin levels, even in the normal population [27]. The estimated frequency of this allele is 0.35 in Caucasians, leading to a homozygous genotype in about 10% of the population, but the frequency is highly variable in different ethnicities [28, 29].

By studying a group of ESRD patients under HD and by using a very broad analytical panel, we were able to demonstrate that higher bilirubin levels, within the normal range (<1 mg/mL), are associated with higher hemoglobin concentration, lower platelet and lymphocyte counts, an increased iron availability, an improvement in the lipid profile, and a decrease in the inflammatory status.

This transversal study showed a prevalence of 8.8% for homozygosity of the TA duplication in the* UGT1A1* promoter region, which is in accordance with the prevalence reported for a healthy Portuguese population [30, 31]. Moreover, we found a relationship between bilirubin levels and the presence of the TA duplication in the promoter region of* UGT1A1*; actually, HD patients homozygous for the (TA)7 allele showed an increase in bilirubin levels, when compared with those heterozygous, or with normal number of TA repeats. It is known that bilirubin is removed during the dialysis process [32], but the* UGT1A1* genotype may modify serum bilirubin levels, even before the next dialysis session, as suggested by the increase in bilirubin levels observed in HD patients homozygous for the (TA)7 allele. When we stratified the results by* UGT1A1* genotype, we only found a significant increase in HDL-c in the group of HD patients homozygous for the (TA)7 allele. Nevertheless, we did not find significant differences in HDL-c when the results were stratified by tertiles of bilirubin, suggesting that this increase in HDL-c in HD patients homozygous for the (TA)7 allele is linked to a decrease in glucuronidation, related to the presence of this polymorphism, more than with the bilirubin serum levels. This association between higher bilirubin concentrations and higher HDL-c was previously described in a large number of studies [23]. Only one study reported decreasing HDL-c concentrations with increasing bilirubin [33].

Stratifying results by tertiles of bilirubin, no significant differences were found in* UGT1A1* genotype distribution between groups, reflecting the low penetrance of the TA duplication polymorphism. Actually, ESRD patients presenting higher bilirubin levels showed also high hemoglobin concentration and hematocrit and erythrocyte counts, suggesting that the red cell mass and hemoglobin concentration are associated with interindividual variations of bilirubin, as previously reported in a healthy population [30, 34] and in individuals with Gilbert's syndrome [35]. High bilirubin levels may be associated also with a decrease in the inflammatory status [36]. Actually, moderate and elevated unconjugated bilirubin concentrations have been associated with a reduced inflammatory status, namely, with lower levels of IL-6 [34, 35]. We also detected an inverse association between bilirubin levels with platelets and lymphocyte counts, suggesting that bilirubin levels may influence hematopoiesis. Recently, it was described that mild elevated serum unconjugated bilirubin levels could delay atherosclerotic plaque progress by preventing thrombus formation through the prevention of collagen induced platelet aggregation [37]. The immunomodulatory effects of unconjugated bilirubin may explain its ability to restrain inflammation [24].

As previously described, high bilirubin levels are associated with low inflammatory grade in nonrenal patients [38, 39] and in ESRD patients [21]. Indeed, a significant decrease in IL-6 levels can be observed in the last tertile of bilirubin. Although we have not found significant differences in CRP between tertiles of bilirubin, a trend towards lower values with increasing bilirubin was found. As previously reported, unconjugated bilirubin is negatively associated with CRP levels [37, 38], which is a widely used biomarker for inflammation status and CVD risk. As IL-6 is a known inducer of hepatic CRP production, the reduction of IL-6 with bilirubin levels could explain, at least in part, the trend towards lower values of CRP concentration in our ESRD patients. It has been suggested that bilirubin is associated with lower CRP levels via reduction of blood lipid concentrations and not by direct inhibition of inflammation [40].

The rise in adiponectin found in HD patients presenting higher bilirubin levels and the significant positive correlation between bilirubin and adiponectin, described here by the first time in HD patients, could justify also the decreased inflammatory markers in HD patients presenting lower bilirubin levels. In fact, several reports demonstrated the anti-inflammatory effects for adiponectin [41–43]. In addition, the predictive value of adiponectin in all-cause mortality in ESRD patients appears to be critically dependent on serum magnesium (s-Mg) and calcium levels (sCa) since strong positive and negative associations of adiponectin with s-Mg and s-Ca were found, respectively, in ESRD patients, and these associations were independent of each other and independent of body composition, nutritional, and inflammatory status [44]. These data are consistent with other studies, which showed a 3% to 10.3% increased risk for all-cause mortality for each 1 mg/mL increment of adiponectin in chronic kidney disease and ESRD patients [45]. Moreover, plasma adiponectin is an independent (inverse) predictor of cardiovascular events and mortality among HD patients. Analysis of adiponectin and several metabolic risk factors have shown that adiponectin has a protective function in prevention of CVD [46]. More recently, it was reported that an increase in obesity-related markers of the metabolic syndrome might be associated with lower adiponectin [47].

The inflammatory stimulus has an important impact in iron metabolism, by mobilizing iron from erythropoiesis traffic to storage sites within the reticuloendothelial system, inhibiting erythroid progenitor proliferation and differentiation [48]. These modifications in iron mobilization lead to an iron depleted erythropoiesis. In this work, the lower grade of inflammation found in the third tertile of bilirubin was associated with an increase in serum iron levels and transferrin saturation and with a decrease in transferrin levels. Additionally, a significant positive correlation between bilirubin and serum iron and transferrin saturation and a significant negative correlation between bilirubin and transferrin were found. We also observed a trend (*P* = 0.108) towards a decrease in hepcidin serum levels that follows the increase in bilirubin levels and the decrease in IL-6 serum levels. The increased iron serum levels were previously reported in Gilbert's syndrome patients [40] similar to our ESRD patients that present higher bilirubin levels. This improvement in iron metabolism seems to be due to the decrease in the inflammatory status that favors iron mobilization from macrophages and increases intestinal iron absorption. Moreover, it has also been hypothesized that bilirubin could induce a mild hemolytic effect, liberating haem, which is subsequently degraded to iron, carbon monoxide and, ultimately, to bilirubin. Carbon monoxide further stimulates haem oxygenase-1 and production/accumulation of bilirubin, inducing further red blood cell lyses, completing the loop of anti-inflammatory compound production [40].

An association between TB and an improvement in the lipid profile has been already described in several studies in nonrenal patients [49–51]. There are evidences that support a role for bilirubin in protecting lipids from various oxygen radical species [52], particularly from lipid peroxidation induced by copper [8]. Indeed, as bilirubin and copper accumulate in atherosclerotic lesions, bilirubin could delay copper induced oxidation of lipids; therefore, the susceptibility to lipid oxidation might be reduced by elevated concentrations of the endogenous antioxidant bilirubin. This could explain the negative relationship between circulating bilirubin and CVD. Moreover, our HD patients presented an association between high bilirubin levels (third tertile) and a decrease in ox-LDL, Apo A, and Apo B and an increase in Apo A/Apo B ratio, in accordance with a previous report [53]. Lipoproteins, particularly LDL-c, are highly susceptible to oxidation, and it is known that the atherogenic process involves an uptake of oxidized LDL by intimal macrophages leading to accumulation of lipid-rich foam cells [54]. Given the antioxidant capacity of bilirubin, it is plausible that bilirubin protects lipids and lipoproteins against oxidation, protecting, therefore, against atherogenesis [15]. Indeed, we found in our HD patients a decrease in ox-LDL/LDL ratio, showing a reduction in LDL oxidation associated with higher bilirubin levels. Considering the known involvement of oxidized LDL in the development of atherosclerosis and the ability of bilirubin to act as a potent lipid chain-breaking antioxidant under physiological conditions, the rise of plasma bilirubin concentrations may reduce the atherogenic risk in HD patients.

We also found an association between higher bilirubin levels and higher PON1 activity. PON1 presents antioxidant properties and contributes to control of the development of oxidative stress at blood level [55, 56]. As referred to, the oxidation of LDL is a crucial starting step for the atherogenic process [57]. By preventing oxidative stress, PON1 contributes to protecting LDL from oxidative modifications, reducing foam cell formation, and inhibiting atherosclerosis [58]. In line, serum antioxidant activity of PON1 is an important factor in cardiovascular diseases, providing protection from oxidative stress and lipid peroxidation [59]. PON1 concentration has been proposed as a predictive marker of cardiovascular mortality and all-cause mortality [60]. Reduced serum PON1 activity has been clearly established in HD patients and could contribute to accelerating the development of atherosclerosis in these patients [61], as they are, probably, more susceptible to the harmful effects of lipid peroxidation than healthy subjects with a normal PON1 activity. An association between PON1 activity after dialysis with creatinine changes, advanced glycation end products, and acrolein has been found, suggesting that uremic toxins could play a mechanistic role in PON1 inactivation [61]. A reduced PON1 activity in HD patients who are not under statin therapy is strongly associated with inflammation, longer time on dialysis, and high recombinant human erythropoietin (rhEPO) doses, suggesting that the reduction in PON1 activity may worsen the prognosis of these patients [62]. Treatment with rhEPO seems to reduce the oxidative stress and to improve PON1 activity in HD patients [63]; patients who are resistant to this therapy present higher levels of inflammatory and oxidative stress markers that could contribute to the lower PON1 activity [64].

In conclusion, this work shows that higher bilirubin levels present potential beneficial effects in ESRD patients under HD, namely, in the prevention and/or development of CVD, by improving lipid profile and reducing the inflammatory grade, which may increase iron availability. Moreover, we described for the first time an association between high bilirubin levels, within the normal range, with higher adiponectin and PON1 activities.

## Figures and Tables

**Table 1:** Results of studied variables (sociodemographic data and dialysis adequacy, *UGT1A1 *genotype, hematological data, iron metabolism, lipid profile, and inflammatory markers) by tertiles of bilirubin.

	All patients (*n* = 191)	Tertiles of total bilirubin				
		T1 TB: <0.21 mg/dL (*n* = 67)	T2 TB: 0.21 to 0.3 mg/dL (*n* = 61)	T3 TB: >0.3 mg/dL (*n* = 63)	*P* value^b^	*P* value^c^
Sociodemographic data and dialysis adequacy						
Age, years	66.1 ± 14.0	67.7 ± 13.6	65.5 ± 15.5	65.1 ± 13.0	0.511	—
Gender (male), *n* (%)	105 (55)	33 (49.3)	31 (50.8)	41 (65.1)	0.142	—
Kt/V urea	1.5 ± 0.3	1.5 ± 0.4	1.5 ± 0.2	1.4 ± 0.2	0.360	0.362
Creatinine, mg/dL	8.1 ± 2.8	7.7 ± 2.4	8.2 ± 2.6	8.5 ± 3.3	0.306	0.407
Urea reduction ratio, %	76.0 ± 6.6	76.2 ± 7.6	76.8 ± 6.2	75.1 ± 5.6	0.377	0.382
Dose of darbepoetin-*α*, *µ*g/kg/week	0.4 (0.2–0.7)	0.5 (0.3–0.8)	0.4 (0.2–0.6)	0.5 (0.2–0.9)	0.311	0.335
Central venous catheter use, *n* (%)	44 (23.0)	20 (29.9)	14 (23.0)	10 (15.9)	0.167	—
BMI, kg/m^2^	25.9 ± 4.6	26.3 ± 5.4	26.1 ± 4.1	25.3 ± 4.2	0.370	0.421

*UGT1A1* genotype						
6/6, *n* (%)	94 (49.2)	33 (49.3)	35 (57.4)	26 (41.3)	0.236	—
6/7, *n* (%)	81 (42.4)	31 (46.3)	20 (32.8)	30 (47.6)		
7/7, *n* (%)	16 (8.4)	3 (4.5)	6 (9.8)	7 (11.1)		

Hematological data						
Hemoglobin, g/dL	11.7 ± 1.4	11.4 ± 1.5	12.0 ± 1.4^a^	11.8 ± 1.3	0.042	0.041
Hematocrit, %	36.4 ± 4.6	35.3 ± 4.5	37.5 ± 4.7^a^	36.6 ± 4.3	0.026	0.022
Erythrocytes, ×10^12^/L	3.8 ± 0.5	3.7 ± 0.5	4.0 ± 0.5^a^	3.8 ± 0.5	0.043	0.042
Reticulocytes, ×10^9^/L	49.3 (27.4–72.8)	56.0 (28.5–75.6)	40.1 (28.3–66.8)	50.8 (26.2–74.7)	0.318	0.325
RPI	0.9 (0.5–1.4)	0.9 (0.5–1.3)	0.8 (0.5–1.4)	1.1 (0.5–1.5)	0.834	0.830
Platelets, ×10^9^/L	183.7 ± 55.1	197.7 ± 59.9	175.8 ± 43.6	164.0 ± 53.6^a^	0.027	0.040
White blood cells, ×10^9^/L	6.4 ± 2.0	6.9 ± 2.0	6.3 ± 2.0	6.0 ± 1.9^a^	0.046	0.057
Neutrophils, ×10^9^/L	4.0 ± 1.5	4.2 ± 1.3	3.8 ± 1.6	3.8 ± 1.6	0.247	0.287
Lymphocytes, ×10^9^/L	1.7 ± 0.7	1.8 ± 0.9	1.7 ± 0.6	1.5 ± 0.5^a^	0.011	0.011
Neutrophil/lymphocyte ratio	2.3 (1.8–3.3)	2.3 (1.8–3.4)	2.2 (1.5–3.0)	2.5 (2.0–3.3)	0.119	0.120

Iron metabolism						
Iron, mg/dL	38.0 (30.0–54.0)	36.0 (29.0–43.0)	41.0 (29.0–55.0)	44.0 (32.0–56.0)^a^	0.005	0.006
Transferrin, mg/dL	184.3 ± 35.6	193.3 ± 34.6	182.4 ± 39.4	176.4 ± 31.1^a^	0.023	0.024
Transferrin saturation, %	17.7 ± 10.8	13.9 ± 6.2	17.7 ± 9.4	21.5 ± 14.1^a^	<0.001	<0.001
sTfR, nmol/L	23.3 ± 11.9	21.7 ± 9.4	25.0 ± 14.2	23.4 ± 11.7	0.281	0.284
Ferritin, ng/mL	402.0 ± 152.6	369.3 ± 153.9	419.4 ± 162.7	419.8 ± 137.0	0.093	0.090
Hepcidin-25, ng/mL	1599.1 (863.6–2409.0)	1738.0 (1144.3–2637.7)	1649.8 (931.7–2440.1)	1476.6 (537.8–2350.0)	0.108	0.101

Lipid profile						
Total cholesterol, mg/dL	154.4 ± 43.4	156.7 ± 34.6	151.3 ± 35.1	155.0 ± 57.3	0.777	0.796
Triglyceride, mg/dL	119.0 (91.0–176.0)	135.0 (91.0–183.0)	113.0 (96.5–172.5)	109.0 (85.0–151.0)	0.156	0.103
HDL-cholesterol, mg/dL	42.3 ± 13.5	41.7 ± 12.1	42.5 ± 13.6	42.7 ± 14.9	0.908	0.888
LDL-cholesterol, mg/dL	73.5 ± 29.5	73.8 ± 28.5	73.2 ± 28.0	73.4 ± 32.2	0.992	0.999
ox-LDL, U/L	36.0 ± 15.4	39.8 ± 21.3	34.5 ± 8.6	33.2 ± 12.0^a^	0.033	0.041
ox-LDL/LDL-c ratio, U/mg	0.053 ± 0.019	0.058 ± 0.025	0.051 ± 0.013^a^	0.048 ± 0.013^a^	0.007	0.005
Lp(a), mg/dL	45.4 (25.2–88.1)	50.4 (27.3–106.2)	45.4 (24.1–89.1)	37.4 (24.7–68.2)^a^	0.052	0.071
Apo A, mg/dL	123.1 ± 30.5	128.9 ± 32.4	125.7 ± 32.7	114.3 ± 23.8^a^	0.017	0.013
Apo B, mg/dL	72.8 ± 21.8	81.8 ± 21.3	70.6 ± 18.9^a^	65.4 ± 31.9^a^	<0.001	<0.001
Apo A/Apo B ratio	1.83 ± 0.67	1.68 ± 0.54	1.89 ± 0.74	1.85 ± 0.65^a^	0.048	0.054

Inflammatory markers						
Adiponectin, mg/L	9.2 ± 4.7	7.9 ± 3.7	9.1 ± 4.6	10.7 ± 5.4^a^	0.003	0.004
PON1, nmol p nitrofenol/mL/min	398.3 ± 92.6	376.0 ± 70.1	398.5 ± 92.9	418.6 ± 106.2^a^	0.040	0.033
CRP, mg/dL	5.2 (2.3–13.3)	7.1 (3.2–14.6)	4.9 (2.1–13.5)	3.6 (1.9–12.7)	0.393	0.453
IL-6, pg/mL	2.3 (1.4–4.3)	3.1 (2.1–5.4)	2.1 (1.4–4.0)^a^	1.6 (1.1–3.4)^a^	0.001	0.003
Albumin, g/dL	3.9 ± 0.4	3.9 ± 0.3	3.9 ± 0.4	3.9 ± 0.4	0.936	0.976

^a^P value < 0.05 for post hoc test considering T1 as reference category; ^b^ANOVA analysis for continuous covariates and Chi-squared test or Fisher's exact test for categorical covariates. ^c^Adjusting for age (ANCOVA). BMI: body mass index; RPI: reticulocyte production index; sTfR: soluble transferrin receptor; HDL: high-density lipoprotein; LDL: low-density lipoprotein; ox-LDL: oxidized low-density lipoprotein; Lp(a): lipoprotein (a); Apo A: apolipoprotein A; Apo B: apolipoprotein B; PON1: paraoxonase 1; CRP: C-reactive protein; IL-6: interleukin-6.
